# Low DICER1 expression is associated with poor clinical outcome in adrenocortical carcinoma

**DOI:** 10.18632/oncotarget.4261

**Published:** 2015-06-11

**Authors:** Gabriela Resende Vieira de Sousa, Tamaya C. Ribeiro, Andre M. Faria, Beatriz M.P. Mariani, Antonio M. Lerario, Maria Claudia N. Zerbini, Iberê C. Soares, Alda Wakamatsu, Venancio A.F. Alves, Berenice B. Mendonca, Maria Candida B.V. Fragoso, Ana Claudia Latronico, Madson Q. Almeida

**Affiliations:** ^1^ Unidade de Suprarrenal, Endocrinologia do Desenvolvimento, Laboratório de Hormônios e Genética Molecular LIM42, Divisão de Endocrinologia e Metabologia, Hospital das Clínicas, Faculdade de Medicina da Universidade de São Paulo, São Paulo, SP, Brazil; ^2^ Instituto do Câncer do Estado de São Paulo, Faculdade de Medicina da Universidade de São Paulo, São Paulo, SP, Brazil; ^3^ Laboratório de Patologia Hepática LIM14, Divisão de Anatomia Patológica, Hospital das Clínicas, Faculdade de Medicina da Universidade de São Paulo, São Paulo, SP, Brazil

**Keywords:** DICER1, TARBP2, miRNA, adrenocortical carcinoma, recurrence

## Abstract

Low DICER1 expression was associated with poor outcome in several cancers. Recently, hot-spot *DICER1* mutations were found in ovarian tumors, and *TARBP2* truncating mutations in tumor cell lines with microsatellite instability. In this study, we assessed DICER1 e TRBP protein expression in 154 adult adrenocortical tumors (75 adenomas and 79 carcinomas). Expression of *DICER1* and *TARBP2* gene was assessed in a subgroup of 61 tumors. Additionally, we investigated mutations in metal biding sites located at the RNase IIIb domain of *DICER1* and in the exon 5 of *TARBP2* in 61 tumors. A strong DICER1 expression was demonstrated in 32% of adenomas and in 51% of carcinomas (*p* = 0.028). Similarly, *DICER1* gene overexpression was more frequent in carcinomas (60%) than in adenomas (23%, *p* = 0.006). But, among adrenocortical carcinomas, a weak DICER1 expression was significantly more frequent in metastatic than in non-metastatic adrenocortical carcinomas (66% vs. 31%; *p* = 0.002). Additionally, a weak DICER1 expression was significantly correlated with a reduced overall (*p* = 0.004) and disease-free (*p* = 0.005) survival. In the multivariate analysis, a weak DICER1 expression (*p* = 0.048) remained as independent predictor of recurrence. Regarding *TARBP2* gene, its protein and gene expression did not correlate with histopathological and clinical parameters. No variant was identified in hot spot areas of *DICER1* and *TARBP2*. In conclusion, a weak DICER1 protein expression was associated with reduced disease-free and overall survival and was a predictor of recurrence in adrenocortical carcinomas.

## INTRODUCTION

Adrenocortical carcinoma (ACC) is a rare neoplasia with an estimated incidence of 0.5–2.0/million/year in adults [1, 2]. There are currently few therapeutic options for patients with ACC, and new insights into the pathogenesis of this lethal disease are needed [1, 3, 4]. In Southern Brazil, the incidence of adrenocortical tumors (ACTs) is remarkably high, being estimated as 10–15 times greater than the worldwide incidence [3]. Overexpression of *IGF2/IGF1R* and constitutive activation of β-catenin were identified as key factors involved in the development of ACC (4–6).

MicroRNAs (miRNAs) are a functional class of noncoding RNA molecules that regulate translation and degradation of messenger RNA. miRNA expression profile of human tumors has been characterized by an overall miRNA downregulation [7, 8]. Recently, several studies demonstrated the potential of miRNA profiling in differentiating between adrenocortical adenomas and carcinomas, risk stratification and prognosis [9]. However, little is known about posttranscriptional regulation of miRNAs. We have recently demonstrated that expression of LIN28, a highly conserved RNA-binding protein that has emerged as a modulator of the processing of *let*-7, was associated with recurrence in ACCs [10]. Interestingly, overexpression of *mir-9*, a negative *LIN28A* regulator, was a significant predictor of poor outcome in ACC patients [10].

DICER1 enzyme and its cofactor, transactivation response (TAR) RNA-binding protein (TRBP), are a key component of the miRNA processing machinery [11, 12]. DICER1, an RNase III endoribonuclease, cleaves double-stranded RNA and pre-miRNA into short double-stranded RNA fragments called small interfering RNA and miRNA respectively. It was demonstrated that escaping miRNA control in cancer cells due to *Dicer* downregulation may allow the phenotypic emergence of more aggressive genetic variants, accelerating breast cancer progression [13]. Recently, *DICER1* mutations in the RNase IIIb domain were found in 29% of nonepithelial ovarian tumors, predominantly in Sertoli–Leydig cell tumors (60%) [14]. Similarly to ACTs, Sertoli–Leydig cell tumors are steroidogenic tumors. These mutations were restricted to codons encoding metal-binding sites within the RNase IIIb catalytic centers, which are critical for miRNA interaction and cleavage. In mouse models of cancer, the loss of a single *Dicer1* allele (haploinsufficiency) reduced the time to tumor onset or survival time [15, 16]. In ovarian cancer patients, low *DICER1* gene expression was significantly associated with advanced tumor stage, poor response to chemotherapy and reduced disease-free survival [17].

Truncating mutations in *TARBP2* gene, encoding the TRBP protein, were identified in sporadic and hereditary carcinomas with microsatellite instability [18]. Two frameshift mutations in *TARBP2* were identified: the deletion of a C in a (C)_5_ coding microsatellite repeat of exon 5 in the colorectal cancer cell line Co115 and the insertion of a C in a (C)_7_ coding microsatellite repeat of exon 5 in the endometrial cancer cell line SKUT-1B. The presence of *TARBP2* frameshift mutations causes diminished TRBP protein expression and a defect in the processing of miRNAs [18]. The reintroduction of TRBP in the deficient cells restores the efficient production of miRNAs and inhibits tumor growth. Most important, the TRBP impairment is associated with a destabilization of the DICER1 protein [18].

The aim of our study was to investigate expression of *DICER1* and *TARBP2* at messenger and protein levels in a large cohort of adult ACTs. Additionally, we investigated mutations in metal biding sites located at the RNase IIIb domain of *DICER1* and in the (C)_5_ coding microsatellite repeat of *TARBP2* exon 5. To further characterize their role in tumor progression and prognosis, we correlated these findings with clinical and histopathological parameters.

## RESULTS

### DICER1 protein expression

**Figure 1:**
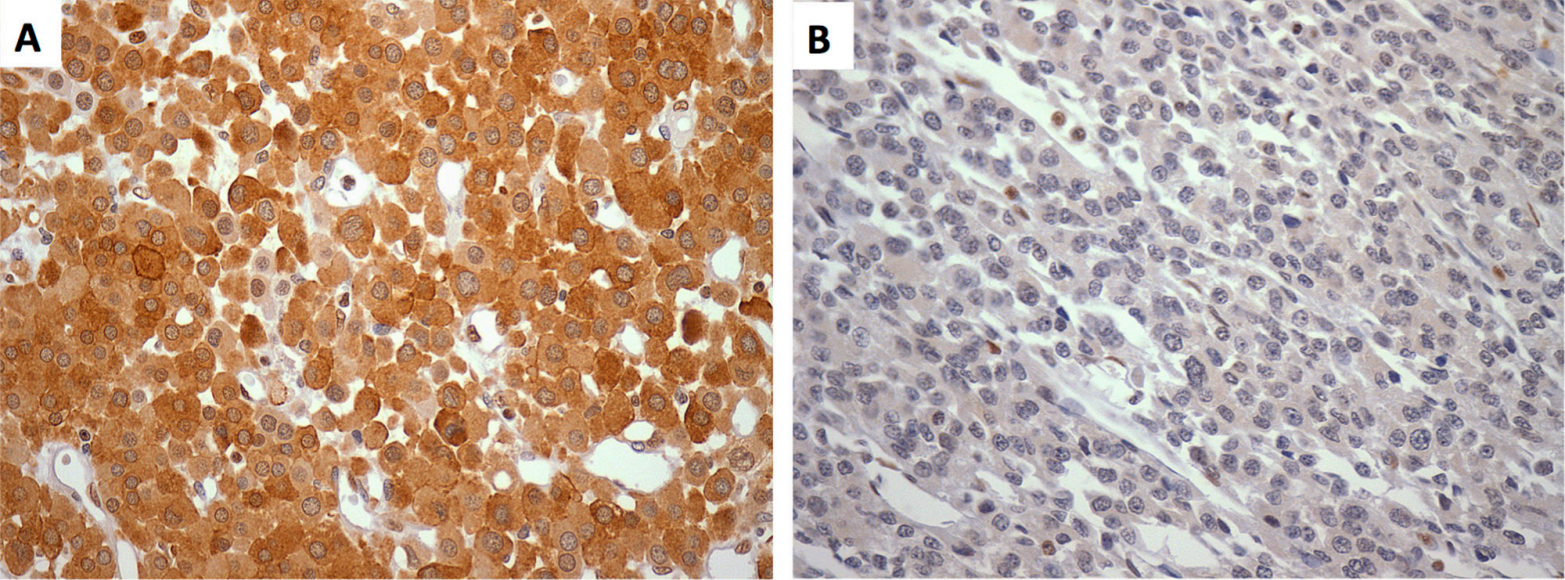
A. Strong imunoreactivity (score 6) for DICER1 in a virilizing ACC in a 59-yr-old woman presenting a favorable outcome after 97 months of follow-up (400x)B. Metastatic ACC in a 30-yr-old man displaying a negative immunoreactivity (score 0) for DICER1 (400x). ACC, adrenocortical carcinoma

In adult ACTs, a strong DICER1 expression was found in 24 out of 75 adenomas (32%) and in 39 out of 79 ACCs (49%; *X^2^* = 4.8, *p* = 0.028) (Figure 1). The median score for DICER1 immunoreactivity was 2.2 (range, from 0 to 6) and 3.5 (0 to 7) for adenomas and ACCs respectively (*p* = 0.001). But, when analyzing only ACCs (*n* = 79), a weak DICER1 expression was significantly more frequent in metastatic than in non-metastatic ACCs (72% *vs.* 38%; *X^2^* = 9,3, *p* = 0.002) (Tables 1 and 2). The time for recurrence after first surgery was 16.3 months (3 to 125) for patients whose tumors displayed weak DICER1 expression and 32.2 months (2 to 376) for those whose tumors displayed strong expression.

**Table 1:** Clinical presentation of adult patients with ACTs

	Adenomas (*n* = 75)	Carcinomas (*n* = 79)
Age (yr)^¶^	41 (15–77)	38 (15–81)
Gender (F:M)	6.5 : 1	3.5 : 1
Follow-up (months)^¶^	68 (12–303)	30 (1–376)
Clinical syndrome *n*		
Cushing	47	13
Virilizing	2	9
Mixed	0	33
Non-functioning	22	16
Others^#^	0	2
Not available	4	5

¶ Median (range);

# Inhibin and estrogen production

ACT, adrenocortical tumor

**Table 2:** Relationship between DICER1 protein expression and baseline clinical or pathological characteristics of 79 adult patients with ACC (only tumor samples derived from primary surgery)

	Weak DICER1 expression	Strong DICER1 expression	*p*
*n* (%)	40 (51%)	39 (49%)	
Score, median	2.4 (0–3.5)	5.4 (3.6–7.0)	
Age, median (yr)	35 (15–67)	45 (18–81)	0.02
Sex [male, n (%)]	11 (28)	6 (15)	0.19
Tumor size, median (cm)	12.3 (6–23)	9.0 (0–23)	0.001
Staging (ENSAT) [n (%)]			0.04
1–2	18 (45)	25 (68)	
3–4	22 (55)	12 (32)	
Hormonal status [*n* (%)]			0.76
Cushing	6 (16)	7 (19)	
Non-Cushing	31 (84)	30 (81)	
Weiss score, median	6 (3–9)	4 (3–9)	0.02
Ki67 index (%)			0.47
<10	25 (63)	26 (70)	
≥10	15 (37)	11 (30)	
Metastasis or local recurrence [n (%)]*			
Affected patients	29 (72)	15 (38)	0.002
Non-affected patients	11 (28)	24 (62)	

ACC, adrenocortical carcinoma

### *DICER1* gene expression

Similarly, *DICER1* gene overexpression was more frequent in ACCs (60%, 15 out of 25) than in adenomas (23%, 7 out of 30; *X^2^* = 7.64, *p* = 0.006). *DICER1* mRNA levels were significantly higher in ACCs (median 3.9, range from 0.37 to 21.3) than in adenomas (1.7, from 0.36 to 23.33; *p* = 0.015). Among ACCs (*n* = 25), *DICER1* gene expression did not correlate with survival, probably because the tumor cohort available for gene expression analysis was smaller than for immunohistochemistry.

In order to investigate the reason for the higher frequency o *DICER1* overexpression in ACCs than in adenomas, we evaluated the expression of *miR-103* and *miR-107* in adrenocortical adenomas and ACCs. It was demonstrated that *miR-103/miR-107* family regulate *DICER1* expression in breast cancer [13]. In our cohort, *miR-103* expression was not significantly different between adenomas (13.7; 6.3 to 62.6) and ACCs (20.3; from 3.0 to 393.4, *p* = 0.37). Regarding *miR-107*, its expression was significantly higher in carcinomas (31.9; 4.6 to 165.8) than in adenomas (17.3; 3.4 a 329.5, *p* = 0.049). Among ACCs, both *miR-103* and *miR-107* expression did not correlate with overall and disease-free survival.

### Survival analysis

**Figure 2:**
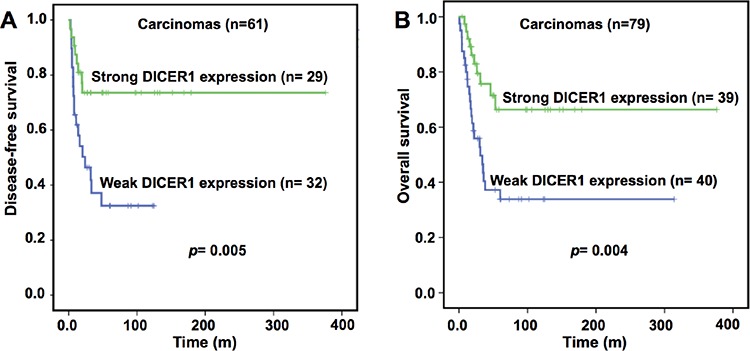
**Impact of DICER1 protein expression on disease-free A. and overall B. survival in adult patients with ACC 79 tumor samples derived from primary surgery with complete clinical data)**. For disease-free survival analysis, only patients with complete resection have been analyzed. ACC, adrenocortical carcinoma.

Interestingly, a weak DICER1 expression was significantly correlated with a reduced disease-free (*p* = 0.005) and overall survival (*p* = 0.004) in ACCs (Figure 2). Regarding disease-free survival, a weak DICER1 expression (*p* = 0.017), a Weiss score ≥ 6 (*p* = 0.001) and a Ki67 index ≥ 10% (*p* = 0.0001) predicted recurrence (local or metastasis). In multivariate analysis, a weak DICER1 expression (HR 2.6, 95% CI 1.1–6.7; *p* = 0.048) and a Ki67 ≥ 10% (HR 6.2, 95% CI 2.5–15.6; *p* = 0.0001) remained as independent predictors of recurrence (Table 3).

**Table 3:** Prognostic factors for overall survival and disease-free survival in adult patients with ACC (only tumor samples derived from primary surgery)

	Univariate analysis			Multivariate analysis		
	Overall survival					
	HR	CI 95%	*p*	HR	CI 95%	*p*
Age	0.99	0.98–1.0	0.87			
Male sex	0.85	0.36–2.0	0.71			
Tumor ≥ 8 cm	24.6	0.3–1778	0.14			
Staging (ENSAT 3/4)	4.8	2.3–9.9	0.0001	2.9	1.3–6.8	0.014
Cushing syndrome	1.0	0.4–2.4	0.99			
Weiss score ≥ 6	4.2	1.9–9.2	0.001	2.0	0.8–5.3	0.14
Ki67 index ≥ 10%	3.9	1.9–8.0	0.0001	2.5	1.2–5.2	0.017
Weak DICER1 expression	2.8	1.3–8	0.006	1.7	0.8–3.3	0.14
	**Disease-free survival**					
	**HR**	**CI 95%**	***p***	**HR**	**CI 95%**	***p***
Age	1.0	0.98–1.0	0.75			
Male sex	1.5	0.7–3.3	0.36			
Tumor ≥ 8 cm	26	0.3–2169	0.15			
Cushing syndrome	0.6	0.2–1.7	0.35			
Weiss score ≥ 6	3.8	1.7–8.6	0.001	1.5	0.6–4.0	0.4
Ki67 index ≥ 10%	6.3	2.8–14.3	0.0001	6.2	2.5–15.6	0.0001
Weak DICER1 expression	3.1	1.4–14.3	0.008	2.6	1.1–6.7	0.048

ACC, adrenocortical carcinoma; HR, hazard ratio

Regarding overall survival, a weak DICER1 expression (*p* = 0.006), ENSAT 3/4 stage (*p* = 0.0001), Weiss score ≥ 6 (*p* = 0.001) and a Ki67 index ≥ 10% (*p* = 0.0001) were associated with reduced overall survival (Table 3). In the multivariate analysis, only ENSAT 3/4 stage (HR 2.9, 95% CI 1.3–6.8; *p=* 0.014) and a Ki67 index ≥ 10% (HR 2.5, 95% CI 1.2–5.2; *p=* 0.017) remained as predictors of reduced overall survival.

### *TARBP2* gene and protein (TRBP) expression

**Figure 3:**
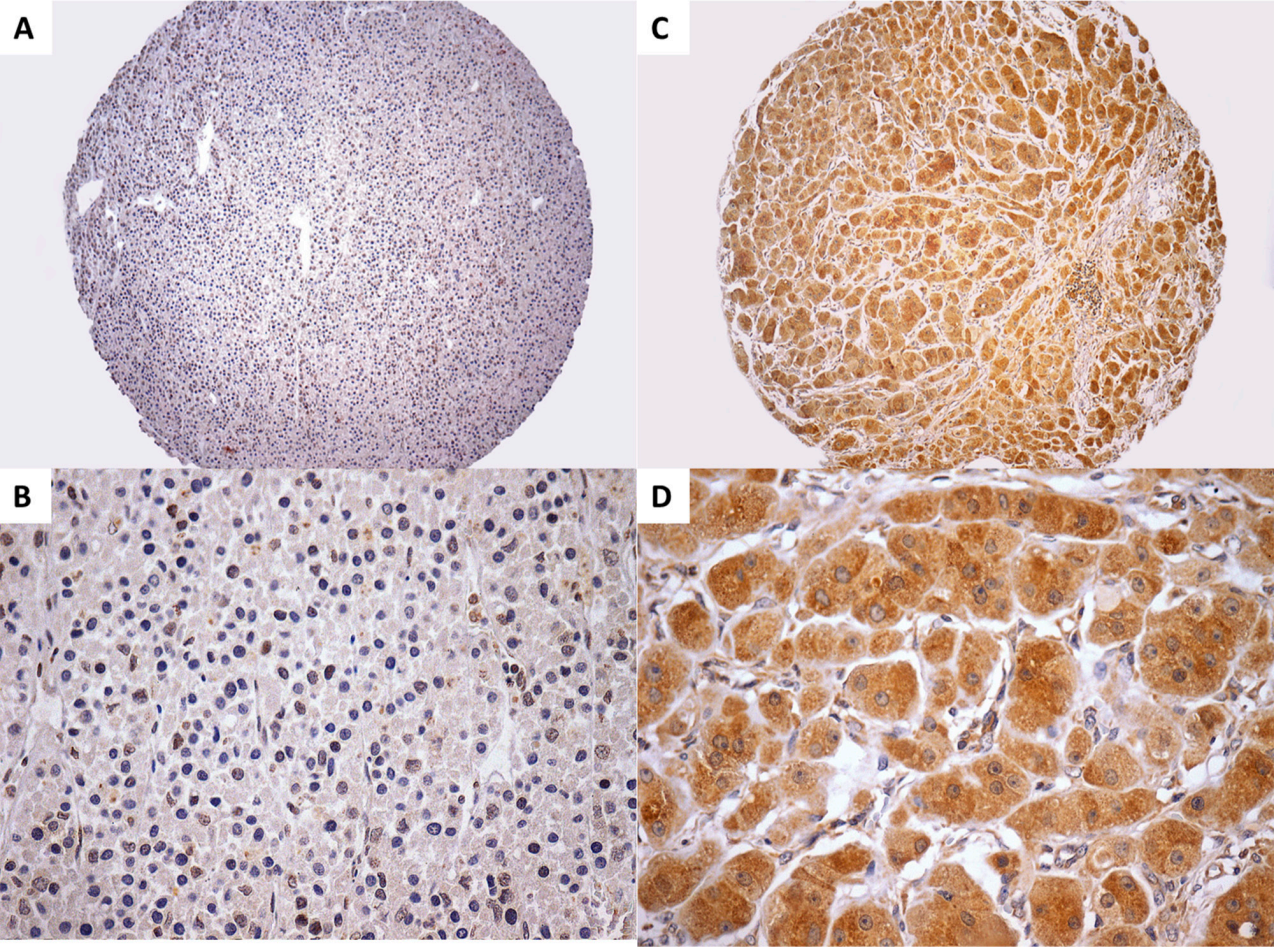
A–B. Adrenocortical adenoma showing a negative immunoreactivity (score 0) for TRBP (A, 100x and B, 400x). C–D. ACC displaying a strong immunoreactivity (score 6) for TRBP (C, 100x and D, 400x). ACC, adrenocortical carcinoma

A strong TRBP expression was identified in 11 out of 73 adenomas (15%) and in 7 out of 80 ACCs (8%; *X^2^* = 1.47, *p* = 0.22) (Figure 3). Then, TRBP expression was not significantly different between adrenocortical adenomas and ACCs. In addition, most of ACTs (88%) displayed a weak TRBP expression. Among ACCs showing a weak TRBP expression, 28 out 73 (38%) ACC patients died during follow-up, whereas 6 out of 7 (86%) ACC patients whose tumors displayed a strong TRBP expression died during follow-up. The time of follow-up for patients whose carcinomas displayed weak and strong TRBP expression was 34 (4.2 to 376.2) and 20.5 (8.8 to 48.7) months, respectively. However, the low number of ACCs with strong TRBP expression precludes any conclusion about survival.

Regarding *TARBP2* gene expression, *TARBP2* mRNA levels were similar between adrenocortical adenomas (median 0.54; range, 0.1 to 9.7) and ACCs (0.52; 0.1 to 2.7, *p* = 0.71). Among ACCs (*n* = 30), *TARBP2* expression did not correlate with overall (*p* = 0.31) and disease-free survival (*p* = 0.85).

### *DICER1* and *TARBP2* sequencing

No genetic variant was identified in metal biding sites located at the RNase IIIb domain of *DICER1* gene and in the exon 5 of *TARBP2* gene in 61 ACTs.

## DISCUSSION

The deregulation of miRNA processing enzymes and their cofactors has been already demonstrated in several types of cancers, suggesting a pivotal role of miRNA processing disruption in tumor progression [19]. In the currenty study, we demonstrated that DICER1 expression was higher in ACCs when compared to adenomas in adults. When analyzing only ACCs, a weak DICER1 expression correlated with a reduced disease-free and overall survival. In the multivariate analysis, a weak DICER1 expression remained as a significant predictor of local recurrence and/or metastasis.

Recently, Caramuta et al. [20] demonstrated DICER1 protein overexpression in ACCs compared to adrenocortical adenomas. However, the small cohort of carcinomas (only 19 ACCs) did not allow any conclusion about the impact of DICER1 expression in ACC patient survival [20]. Here, we showed that a weak DICER1 expression was associated with larger tumor size, more advanced staging and higher Weiss score. Then, our findings are pioneer in demonstrating that recurrent ACCs present a significant reduction in DICER1 protein expression.

In agremment with our results, a weak DICER expression has been associated with poor outcome in several malignancies [19]. A low DICER protein expression evaluated by immunohistochemistry was associated with advanced tumor stage, poor response to chemotherapy and reduced disease-free survival in ovarian cancer patients [21]. In gastric cancer, a low DICER1 staining was a predictor of local linfonodal invasion [22]. Similarly, a weak or negative DICER1 expression correlated with reduced overall and disease-free survival in other malignancies, such as colon cancer [23] and gallbladder cancer [24].

Based on our findings, we can speculate that DICER1 overexpression at mRNA and protein levels in ACCs compared to adenomas can represent a compensatory event due to deregulation of miRNA machinery components in malignant tumors. The *miR-103/miR-107* family is known to regulate *DICER1* expression in breast cancer [13]. Here, we demonstrated that *miR-107* was overexpressed in carcinomas when compared to adenomas. Then, we can hypothesize that *miR-107* overexpression might explain *DICER1* expression in ACCs. Indeed, *miR-107* overexpression has been demonstrated in several cancers, such as colon, stomach, pâncreas and esophagus [25, 26].

Although the lack of correlation between *DICER1* gene expression and overall survivor in ACC may be due to the small size of the cohort available for gene expression analysis, the occurrence of post-translational events be responsible or contribute for this finding. Recently, Gross et al. [27] demonstrated DICER1 SUMOylation by the UBC9 enzyme, which promotes DICER1 post-translational degradation.

In this study, the loss of DICER1 protein expression was a molecular predictor of local recurrence and/or metastasis. Although at face value these data seem contradictory, we can rationalize them by the fact adrenocortical adenoma development and ACC progression are not a continuous process [2]. Additionaly, we could not conclude if the loss of DICER1 protein expression has a direct role in cancer progression or is an epiphenomenon reflecting underlying abnormalities. In other cancers, low expression of DICER1 has been associated with inactivating *DICER1* mutations and *miR-107/103* deregulation [13, 14]. We have ruled out both hypothesis to explain the loss of DICER1 expression in metastatic ACC, but other miRNAs can possibly be involved in DICER1 downregulation.

It was recently shown that hot-spot *DICER1* mutations were highly prevalent in Sertoli–Leydig cell tumors [14]. *DICER1* hot-spot mutations were also found in a single high-grade ovarian sarcoma, one testicular germ-cell and two embryonal rhabdomyosarcomas [14]. Since Sertoli–Leydig cell tumors are steroidogenic tumors, it prompted us to investigate DICER1 hot-spot mutations in ACTs. We did not find mutations in the in metal biding sites located at the RNase IIIb domain of *DICER1* gene, showing that the loss of DICER1 protein expression was not caused by inactivating mutations. In the mutation database of the Catalogue of Somatic Mutations in Cancer (COSMIC), only 4 of 938 cancers have somatic mutations outside of RNase IIIb hot spots [28]. Based on this evidence, we decided not to sequence the entire gene besides the RNase IIIb domain.

TRBP is an integral component of a DICER1-containing complex, interacts directly with the DICER1 protein and is required for the stabilization of the DICER1 protein [29, 30]. In addition, Melo et al. [18] identified *TARBP2* truncating mutations in sporadic and hereditary carcinomas with microsatellite instability. Recently, TRBP overexpression was demonstrated in ACCs and *TARBP2* gene expression was useful to discriminate between adrenocortical adenomas and carcinomas [20]. In contrast to this previous data, *TARBP2* gene and protein (TRBP) expression was not different between adrenocortical adenomas and ACCs in our cohort of ACTs. Since we had only 7 ACCs displaying a strong TRBP expression, we could not reach any conclusion about the impact of TRBP expression on survival of ACC patients.

In conclusion, *DICER1* gene and protein expression was higher in ACCs than in adenomas. But, among ACCs, a weak DICER1 protein expression was significantly associated with reduced disease-free and overall survival. Additionally, a weak DICER1 protein expression was an independent predictor of recurrence in ACC patients.

## PATIENTS AND METHODS

The study was approved by the Ethics Committees of the Hospital das Clínicas, University of São Paulo and informed written consent was obtained from all patients. The Weiss criteria were used to classify adenomas and carcinomas (Weiss score < 3 and ≥ 3, respectively). DICER1, TRBP and Ki67 protein expression was assessed in a total of 154 ACTs (75 adenomas and 79 carcinomas) (Table 1). Among them, 61 ACTs (31 adenomas and 30 carcinomas) were used to analyze *DICER1* and *TARBP2* gene expression and DNA sequencing.

All tumors samples derived from primary surgery. Clinical parameters, such as sex, age at diagnosis, date of surgery, tumor size, pathological classification, and hormone analysis were collected from patient records. Tumor stage was classified according to the European Network for the Study of Adrenal Tumors (ENSAT) classification. Only patients with at least 12 months of follow-up were included in this study. Presence of distant metastases or recurrence was evaluated at the time of diagnosis and during follow-up visits by computerized tomography of chest and abdomen every 3–6 months.

### Tissue microarray (TMA) and immunohistochemical analysis

Representative areas of the ACTs (viable tumor tissue without necrosis) were identified on hematoxylin- and eosin-stained slides and marked on paraffin donor blocks. The spotted areas of donor blocks were punched (1.0 mm punch) and mounted into 3 recipient paraffin blocks using a precision microarray instrument (Beecher Instruments, Sun Prairie, WI). One set of three slides was selected (one slide from each of the 3 TMA paraffin-blocks of the triplicate) for staining with anti-DICER1 rabbit polyclonal antibody (titer 1:100; HPA 000694*, Sigma Life Science, St. Louis, United States*) and anti-TRBP rabbit polyclonal antibody (ab72547, *Abcam, Cambridge, United Kingdon*). An immunoperoxidase immunohistochemical modified method with humid heat antigen retrieval was used as previously described [31]. DICER1 immunostaining was blindly evaluated by two independent observers (I.C.S. and M.C.N.Z) and the mean of the two evaluations was taken for statistical analysis. The inter-observer agreement coefficiente (Kappa) for DICER1 staining evaluation was 0.72 (*p* < 0.001). A kappa coefficient > 0.61 is considered substantial agreement. TRBP staining was evaluated by the pathologist M.C.N.Z. The positive control for anti-DICER1 was normal gastric mucosa and for anti-TRBP was normal adrenal cortex.

TMA samples were included in the analysis only if two or more evaluable cores were available after the staining procedure. Cytoplasmic staining was evaluated according intensity as negative (0), low (1), medium (2), or strong (3). The percentage of positive tumor cells was visually scored as follow: 0 if 0% of tumor cells were positive; 1 if 1–25%; 2 if 26–50%, 3 if 51–75% and 4 if 76–100%. A semiquantitative score was then calculated by sum of the staining intensity with the proportion score with a final score ranging from 0 to 7. The median score was a priori chosen as cut-off point for separating tumors with low and strong staining.

Another set of three slides was stained with mouse monoclonal anti-human Ki67 antigen (titer 1:40, clone MIB-1, code M7240, Dako, Denmark). Nuclear staining for Ki67 in our cohort was performed as previously described [10].

### Quantitative real-time RT-PCR (qRT-PCR)

After surgical resection, tumor fragments were immediately frozen in liquid nitrogen and stored at − 80°C until total RNA extraction using the Trizol reagent (Invitrogen, Carlsbad, CA). RNA samples were treated with DNAse using standard procedures. cDNA was generated from 1 μg of total RNA using the commercial kit Superscript III First Strand S (Invitrogen, Carlsbad, CA). Quantitative real-time PCR was performed in the ABI Prism 7000 sequence detector using TaqMan gene expression assays according to the manufacturer's instructions (Applied Biosystems, Carlsbad, CA). The PCR cycling conditions were as follows: 2 min at 95°C, 40 cycles of 95°C for 15 sec and 60°C for 30 sec, and a final step at 72°C for 30 sec. The assays for target genes were *DICER1* (Hs00998580_m_1_) and *TARBP2* (Hs00998379_m_1_). β-actin (*ACTB*, 4310881E) and β-glucoronidase (*GUSB*, 4310888E) were used as endogenous genes for normalization.

For measuring miRNAs expression, single-stranded cDNA was synthesized from 1 mcg of total RNA using Megaplex RT Primers, Human Pool A v2.1 (PN 4399966, Applied Biosystems) and TaqMan MicroRNA Reverse Transcription Kit (PN 4366596, Applied Biosystems). PCR products were amplified using Taqman Universal Master Mix II, no UNG (PN 4440040, Applied Biosystems). The following TaqMan MicroRNA Assays were used: *miR-103* (000439) and *miR-107* (00443). RNU44 (001094) and RNU48 (001006) were used as endogenous genes for normalization. The cut-off value for overexpression was the median value of all samples for each gene.

### Mutation analysis

DNA from primary tumors was extracted and amplified by PCR using standard procedures. PCR products were sequenced in an automated ABI Prism 3700 sequencer (Applied Biosystems). Metal biding sites located at the RNase IIIb domain of *DICER1* were sequenced as previously described [14]. The *DICER1* primers and their genomic locations are described in Supplementary Table 1. The exon 5 of the *TARBP2* gene was sequenced as previously described [18]: primer forward, 5′CGGGAGATGGTAGTCAGGAA3′; primer reverse: 5′CATCCTCATTTCCCATGCATG3′. The exon 5 contains 3 microsatellite regions: a (C)_7_ coding microsatellite repeat and two (C) coding microsatellite repeats.

### Statistical analysis

Statistical analysis was performed using SPSS Software (22.0; SPSS Inc., Chicago, IL). Continuous data are expressed as median values (from minimum to maximum). Differences in expression levels between two groups were analyzed by means of the two-tailed Mann-Whitney *U* test. One-way ANOVA model followed by Bonferroni *post-hoc* test was employed when comparing three groups. The Fisher's exact test or the χ^2^ test was used to investigate dichotomous variables. Overall survival was defined as the time from the date of primary diagnosis to death related to ACC or last follow-up. Disease-free survival was defined as the time from the date of complete tumor resection to the first radiological evidence of disease relapse or death. All the survival curves were obtained by Kaplan-Meier estimates, and the differences between survival curves were assessed by the log-rank (Mantel-Cox) test. Predictive factors of prognosis were identified by means of Cox proportional hazards regression models, which was used to estimate hazard ratios (HR) and their 95% confidence intervals in univariate and multivariate analysis. *P* < 0.05 was considered significant.

## SUPPLEMENTARY TABLE


