# Supplementary material for: Low DICER1 expression is associated with poor clinical outcome in adrenocortical carcinoma
Source: Oncotarget. 2015 Jun 11;6(26):22724–33. doi: 10.18632/oncotarget.4261 (PMC4673194; doi:10.18632/oncotarget.4261)
Supplement: Supplementary file 1 [file oncotarget-06-22724-s001.pdf]

SUPPLEMENTARY TABLE

Supplementary Table 1: Primers used to sequence the RNaseIIIb domain of the *DICER1* gene

Primer	Sequence	Metal binding sites of the RNaseIIIb domain
Exon23_Forward	TGTAAAACGACGGCCAGTCTTCTGCACAAGCTTACGGTTCCA	E1705 e D1709
Exon23_Reverse	CAGGAAACAGCTATGACCCAGCGATGCAAAGATGGTGTTGT	E1705 e D1709
Exon24_Forward	TGTAAAACGACGGCCAGTGAAACTACATCTGTGGACTGCCTG	E1810 e D1813
Exon24_Reverse	CAGGAAACAGCTATGACTTAGTGGCCGCATCATGGGATAGT	E1810 e D1813
